# Distribution and Prognostic Significance of Estrogen Receptor *α* (ER*α*), Estrogen Receptor *β* (ER*β*), and Human Epidermal Growth Factor Receptor 2 (HER-2) in Thyroid Carcinoma

**DOI:** 10.1155/2020/6935724

**Published:** 2020-05-05

**Authors:** Anjali Mishra, Niraj Kumari, Chandan Kumar Jha, Raouef Ahamed Bichoo, Shravan Kumar Mishra, Narendra Krishnani, Saroj Kanta Mishra

**Affiliations:** ^1^Department of Endocrine Surgery, Sanjay Gandhi Postgraduate Institute of Medical Sciences, Raebareli Road, Lucknow 226014, India; ^2^Department of Pathology, Sanjay Gandhi Postgraduate Institute of Medical Sciences, Raebareli Road, Lucknow 226014, India

## Abstract

**Purpose:**

The primary aim of this study was to determine the incidence of estrogen receptor *α* (ER*α*), estrogen receptor *β* (ER*β*), and human epidermal growth factor receptor 2 (HER-2) expression in various subtypes of thyroid carcinoma (TC) of follicular origin and the secondary aim was to correlate the expression with various clinicopathologic prognostic factors.

**Methods:**

Immunohistochemistry analysis was performed on archival paraffin-embedded tissue sections (1991–2016). ER*α*, ER*β,* and HER-2 expressions were correlated with clinicopathologic prognostic factors, disease recurrence, and overall survival (OS).

**Results:**

A total of 264 TC patients were included in the study. Incidences of ER*α*, ER*β,* and HER-2 were 8.1 vs 16.3 vs 13.9% (*p*=0.15), 26.6 vs 11.5 vs 36.1% (*p*=0.002), and 12.9 vs 2.9 vs 0% (*p*=0.003) in papillary thyroid carcinoma (PTC), follicular thyroid carcinoma (FTC), and poorly differentiated thyroid carcinoma (PDTC), respectively. Overall ER*α* had significant correlation with distant metastases (0.038) and in case of PDTC with multicentricity (*p*=0.037). ER*β* had significant correlation with lymph node metastases (*p*=0.023) in FTC. HER-2 correlated with tumor size (*p*=0.027) only on univariate analysis. OS did not correlate with expression of any receptor.

**Conclusion:**

ER*α*, ER*β,* and HER-2 have differential expression and prognostic implications in different TC subtypes.

## 1. Introduction

There is undeniable association of thyroid carcinoma (TC) with female gender. But only few studies have examined the role of estrogen receptors (ER) in TC [1]. There has been recent rekindling of interest in the subject and experimental studies are trying to look into the mechanisms by which the female sex hormone works on TC cells [2, 3]. Estrogens play a critical role in endocrine tumors, including those of the breast, prostate, and thyroid [4]. As is true for the other malignancies, both isoforms of ER, alpha (ER*α*) and beta (ER*β*), are expressed in TC. ER*α* is linked with estrogen-dependent proliferation and ER*β* with apoptosis and other suppressive functions in thyroid tumors [5]. Presence of ER is routinely looked for in the breast cancer, which is the most common malignancy among women. Another receptor routinely examined in breast cancer is human epidermal growth factor receptor 2 (HER-2) [6]. There is evidence of cross-talks between ER and HER-2 pathways [1]. Presence of ER and HER-2 is of prognostic and therapeutic value in breast cancer, and drugs are available to target these receptors [7]. There is ever growing need to look for alternative therapy in cases of radioiodine refractory TC. Currently, only a few targeted therapies are available, but their efficacy is limited and these are associated with high incidences of debilitating side effects. ER and HER-2 are potential targets, which could be exploited, but there is no enough data on incidence of ER and HER-2 in TC. Therefore, we do not know for sure what proportion of patients would benefit from anti-ER and anti-HER-2 therapy [3, 5, 8–10]. The primary aim of this study was to determine the incidence of ER*α*, ER*β,* and HER-2 expression in various subtypes of TC of follicular origin and the secondary aim was to correlate the expression with various clinicopathologic prognostic factors.

## 2. Materials and Methods

This is a retrospective study (1991–2016). The Institute Research Committee and the Ethics Committee approved the study (2014-187-IMP-EXP). One hundred and twenty-four papillary thyroid carcinoma (PTC), 104 follicular thyroid carcinoma (FTC), and 36 poorly differentiated thyroid carcinoma (PDTC) patients were included in the study. Immunohistochemistry analysis was performed on archival paraffin-embedded tissue sections. Patients with at least 2 years of follow-up were included and those with insufficient data and nonavailable or poorly preserved specimens were excluded from the study. The clinicopathologic profile and follow-up findings were entered in a predesignated proforma.

### 2.1. Immunohistochemistry (IHC)

Thyroid tissue sections were obtained from archives of pathology department of our institute. Paraffin-embedded 4 *μ*m tissue sections fixed in formalin were used. All histology slides were meticulously reviewed by 2 pathologists experienced in thyroid pathology. The diagnosis of thyroid cancer was reconfirmed. PDTC was diagnosed as per Turin criteria [11]. Slides were deparaffinized, rehydrated, and subjected to microwave heat antigen retrieval in 10 mM citrate buffer (pH 6.0) or Tris EDTA (pH 9.0) for 20–25 min. After blocking endogenous peroxidase activity, sections were incubated with primary antibodies against ER*α*, ER*β,* and HER-2 for 2 hours at room temperature. After three washes with PBS, slides were incubated with universal secondary antibody for 30 minutes at room temperature. Immunoreactivity was visualized using the chromogen 3,3′-diamino-benzidine (DAB) and counterstained with hematoxylin.

Cases of breast cancer with positive staining of all the three primary antibodies were used as positive controls. Sections incubated without primary antibodies served as negative controls. Ten cases each of follicular adenoma (FA) and multinodular goiter (MNG) were also included as controls. Two pathologists independently examined the slides. Nuclear positivity of ER*α* and ER*β* was taken as positive stain. Immunostained slides were scored using the Allred scoring system [12]. A combined score of three or higher was defined as positive staining. HER-2 staining was scored according to the updated ASCO-CAP system [13]. 2+ and 3+ scores of complete membranous staining for HER-2/neu were considered positive.

### 2.2. Definitions and Standard

- Metastases: synchronous distant metastases are defined as the metastases detected preoperatively or within 6 months of surgery. Metastases detected 6 months after surgery are termed as metachronous metastases.
- Lymph node metastases: involvement of any cervical level from I to VI.
- Extrathyroidal invasion: gross and/or microscopic invasion.
- Recurrence: it is defined as elevated serum Tg or anti-Tg antibody with or without structural or RAI scan evidence of disease 6 months from the date of surgery.
- Overall survival (OS): day of the surgery was taken as reference point to calculate disease specific survival (OS) and disease-free survival (DFS).

Expression was correlated with subtypes of TC and with various prognostic factors of age, sex, tumor size, lymph nodal status, extrathyroidal invasion, and presence of distant metastases and also with disease recurrence and OS. Data analysis was performed on SPSS software. Descriptive statistics was used according to the distribution of variables. The chi square test or Fisher's exact test was used for the comparison of the frequency or proportions of single variables. Univariate and multivariate analyses were performed to correlate the expression of various IHC markers with the different prognostic factors. Multivariate analysis (step-wise regression with backward selection method) was performed for all the tumors together and each histology type separately. All the prognostic factors selected for the univariate analysis were included and then excluded in the step-wise fashion. The Kaplan–Meier method was used for survival analysis. *p* values less than 0.05 were considered statistically significant for all the tests.

## 3. Results

A total of 264 patients were included in the study. These consisted of 124 PTC, 104 FTC, and 36 PDTC patients. Mean age of the whole cohort was 46.1 ± 15.5 years and the male to female ratio was 1 : 1.9. The mean tumor size was 4.7 ± 2.8 cm. Tumor multicentricity, extrathyroidal invasion; lymph node metastases, and distant metastases were noted in 26.5, 24.6, 38.6 and 31.4% of the patients, respectively. Seventeen percent of the PTC and 66% of FTC specimens showed presence of poorly differentiated areas. Mean follow-up duration was 73 months. Eleven percent patients developed locoregional recurrence, 7% patients developed metachronous distant metastases, and 17% patients died during the follow-up. Ten percent patients were lost to follow-up and were excluded from survival analysis.

Overall incidences of ER*α*, ER*β,* and HER-2 expression were 12%, 22%, and 7%, respectively. None of FA and MNG sections showed ER*α*, ER*β,* and HER-2 expression. Incidences of ER*α* expression (8.1% vs 16.3% vs 13.9%, *p* = 0.15) did not vary significantly among PTC, FTC, and PDTC, whereas ER*β* expression was significantly low in FTC (26.6% vs 11.5% vs 36.1%, *p* = 0.002) and HER-2 expression significantly high in PTC than FTC and was altogether absent in PDTC (12.9% vs 2.9% vs nil, *p* = 0.003). Among HER-2 expressing patients (*n* = 19), eight showed 3+ (overall 3%) and eleven 2+ staining pattern (overall 4%). Nine patients (3.4%) showed 1+ staining. Cytoplasmic staining was observed in 14 (5.3%) patients, 4 of which in addition showed 1+ expression and remaining exclusive cytoplasmic staining. The incidence of cytoplasmic staining was highest in PDTC (8%) followed by PTC (5.6%) and FTC (3.8%).

**Figure 1:**
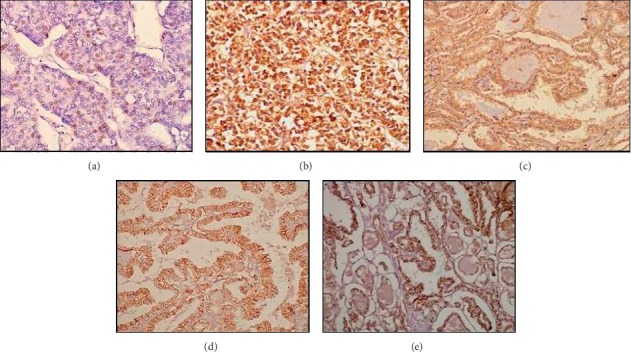
Photomicrographs depicting intranuclear immunostaining of ER*α* in PTC (a), ER*β* in PTC (b), and ER*β* in PDTC (c) and membranous immunostaining of HER-2 in PTC (d) and FTC (e).

There was no correlation among ER*α*, ER*β,* and HER-2 expression (*p* = 0.64, 0.49, and 1.00). Both ER*α* and ER*β* receptor positivity was observed in 6 tumors (PTC = 3, FTC = 2, and PDTC = 1). Among HER-2 expressing tumors, ER*α* and ER*β* expression was observed in one and four patients each of FTC and PTC, respectively, and remaining all had exclusive HER-2 expression. None of the specimen showed all the three receptor expression together. Expression did not correlate with tumor subtypes, i.e., minimal invasive versus widely invasive FTC and PTC variants. The ER*α*, ER*β,* and HER-2 expression pattern was similar in differentiated and poorly differentiated areas of a tumor.  Figure 1 shows the photomicrographs depicting immunostaining for ER*α*, ER*β,* and HER-2 in different TC.

**Figure 2:**
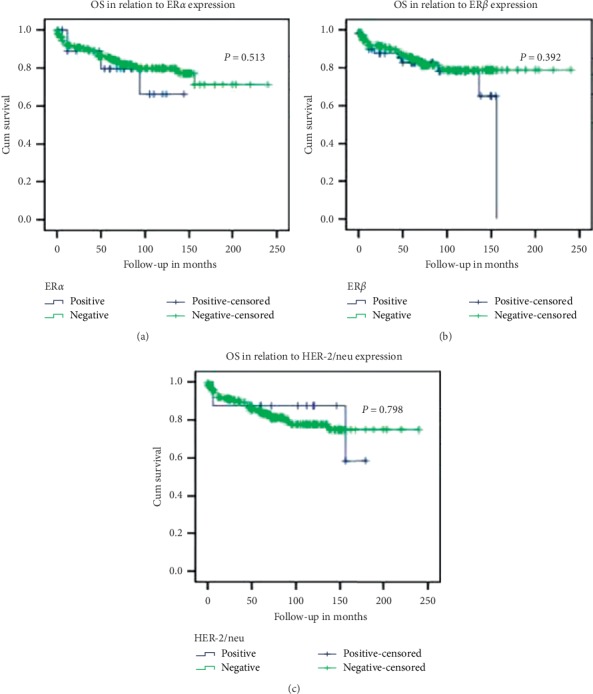
Overall survival in relation to (a) ER*α*, (b) ER*β,* and (c) HER-2 expression.

Clinicopathologic correlation of ER*α*, ER*β,* and HER-2 is summarized in Tables 1–3, respectively. On univariate analysis including all TC, ER*α* expression was significantly associated with distant metastases (*p*=0.040) and ER*β* with lymph node metastases (*p*=0.014) and with presence of poorly differentiated areas (*p*=0.026) within a tumor. HER-2 expression had significant positive correlation with tumor size (*p*=0.027). On analyzing the individual tumor types separately, ER*α* expression significantly correlated with male gender (*p*=0.043) in PTC and multicentricity in PDTC (*p*=0.029), whereas ER*β* with female gender in PDTC (*p*=0.014) and HER-2 with tumor size in FTC (*p*=0.014). On multivariate analysis (Table 4) including all the TC, ER*α* expression was associated with distant metastases (0.038). On separate multivariate analysis for each histology type, no significant correlation was found in case of PTC, whereas significant negative correlation was seen between ER*β* and lymph node metastases (*p*=0.023) in FTC and positive correlation of ER*α* and multicentricity in PDTC (*p*=0.037). None of the receptor expression had any significant correlation with disease recurrence and OS (Figure 2).

## 4. Discussion

The current study shows that ER*α*, ER*β,* and HER-2 are expressed differentially in TC with the least overlap in expressions. ER*α* expression was significantly associated with distant metastases in TC in general and multicentricity in PDTC.

Incidence of ER expression in the current study was at par with other published reports, which ranged from 17–54% [1, 3, 5, 14–17]. ER*α* expression has been correlated with tumor virulence, large tumors, extrathyroidal invasion, distant metastases, and reduced DFS [4–17]. But on the contrary, there are reports, which showed that ER*α* and cosecretary proteins expression are positively associated with well-differentiated tumors and inversely with disease recurrence [1, 3]. In the current study, a significant correlation of ER*α* expression was noted with distant metastases and multicentricity, but not with tumor recurrence and OS. On the other hand, ER*β* expression is generally observed in incidental or less virulent tumor [18, 19]. There are reports of differential expression of ER*α* and ER*β,* but the comparison has generally been available between PTC and PDTC and/or anaplastic carcinoma, and FTC has not been studied much [5]. In the current study, ER*β* expression was significantly more in PTC and PDTC than in FTC.

There are limited but conflicting reports on HER-2 expression in TC. HER-2 expression is generally not seen in normal thyroid tissue and a few studies did not find any significant variation in expression among benign or malignant thyroid tumors [10, 20]. The reported incidence of HER-2 expression varies from 7–18% in PTC [1, 6, 8–10]. There are a few reports showing that the incidence of HER-2 expression is higher in FTC (44–50%), but these had limited number of such cases included in the study [8, 9]. On the contrary, one study reported no HER-2 expression in FTC and an incidence of 6.9% in PTC [21]. Our findings seem more in line with the latter. Expression of the HER-2 has been positively linked with capsular invasion and disease recurrence and inversely with well-differentiated tumors [8–10]. In our study, the expression correlated with tumor size.

The variability in the results of ER and HER-2 expression can partly be explained by the different techniques of detection employed in the various studies. But it is possible that expression of other genes and coregulators might modulate the expression and function of the ER resulting in confounding observations [1, 3]. There is evidence that estrogen may exert a direct effect on thyroid tumorigenesis by ER-independent mechanisms through modulation of the genes involved in cell proliferation independently of its classic nuclear receptor [1, 22–28]. The proliferative effects of estrogen in thyroid cancer are mainly mediated through the regulation of bcl-2, Bax, and c-fos genes [29, 30]. Further, central to the functioning of ER are the coregulators such as steroid receptor coactivator (SRC-1) and nuclear receptor corepressor (NCoR). SRC-1 is significantly associated with the expression of ER*α* but inversely associated with capsular invasion and positively with well-differentiated tumors [1]. In an experimental study, treatment with estrogen increased protein expression of SRC-1 and its target gene cyclin D1 in the FTC-133 cell line and decreased expression of ER*β*, but no alterations in ER*α*, NCoR, or HER-2 protein expression was detected. These effects seem to be contradictory in nature and have partly been explained by the opposing roles of SRC-1 and NCoR [1]. The probable explanation of the contradiction observed in the current study is that no expression of HER-2 in PDTC and yet positive correlation of ER*β* with occurrence of PDTC in females could also be hidden in the presence/absence of such coregulators of ER. Since we did not analyze this aspect in the current study, we cannot be absolutely sure and should wait for the future research to throw light on this subject.

As is true about ER expression, other genetic and epigenetic factors might be responsible for variability in the results of HER-2 expression in TC and SRC-1 is one of them [31]. Another fact worth mentioning is cytoplasmic staining for HER-2, which was noted in 8% of PDTC in the current study. Cytoplasmic staining is usually considered nonsignificant, but in colon cancer, it has been found to have prognostic significance [32]. The exact molecular mechanism behind cytoplasmic expression of HER-2 is yet to be elucidated, but one of the theories is that upregulation of the promoter-binding proteins leads to increase in HER-2 production, which in addition to cell membrane is also expressed in the cytoplasm. Currently, anti-HER- 2 molecule targeting membranous (e.g., trastuzumab) as well as cytoplasmic HER-2 (lapatinib) are available for clinical use [33].

The potential clinical application of ER and HER-2 is therapeutic. Estrogen receptor modulators and targeted therapy against HER-2 are an integral part of already well-established treatment protocols in breast and prostate cancer [6, 7]. A selective estrogen receptor modulator has been used in certain benign thyroid condition, e.g., Reidel's Thyroiditis, and there are some anecdotal reports of the beneficial role of Tamoxifen in multidrug-resistant thyroid cancers [34]. But, currently, there is no robust data to support the use of these medicines in TC. Their utility needs to be explored further in cases of radioiodine-resistant TC.

The strength of the study is that it included large number of cases with various tumor types including the largest number of FTC patients. The limitation is that it was done on archival tissue and expression of various receptors could be under represented. We also did not employ FISH to confirm HER-2 expression in tumors exhibiting a 2+ staining pattern.

## 5. Conclusion

ER*α*, ER*β,* and HER-2 expressions seem to have differential expression and prognostic significance in various thyroid carcinoma subtypes. Their therapeutic implications need to be explored.

## Figures and Tables

**Table 1:** Clinicopathologic correlation of ER*α*.

Sl no.	Attribute	ER*α* positive (*n* = 32)	ER*α* negative (*n* = 232)	*p* value^a^ overall	*p* value^a^ PTC	*p* value^a^ FTC	*p* value^a^ PDTC
1.	Age in years (mean ± SD)	49.2 ± 15.1	45.8 + 15.5	0.24	0.654	0.347	0.199
2.	Gender (M : F)	11 : 31	79 : 153	1.00	0.043^*∗*^	0.229	0.088
3.	Presence of PDA^b^, *n* (%)	11 (40.7)	79 (39.3)	0.33	0.970	0.590	—
4.	Tumor size in cm (mean ± SD)	5.2 ± 3.1	4.6 ± 2.8	1.00	0.210	1.000	0.862
5.	Multicentricity, n (%)	8 (25.0)	62 (27.4)	0.83	0.524	1.000	0.029^*∗∗*^
6.	Extrathyroidal extension, *n* (%)	7 (21.9)	58 (25.4)	0.82	0.451	0.669	1.000
7.	Lymph node metastases, *n* (%)	12 (37.5)	90 (39.0)	1.00	0.739	1.000	0.630
8.	Distant metastases, *n* (%)	15 (48.4)	68 (29.4)	0.04^*∗∗∗*^	1.000	0.062	1.000
9.	Recurrence, *n* (%)	2 (6.2)	23 (9.9)	1.00	1.000	1.000	1.000
10.	Mean survival in months (*n* ± SE)	114 ± 11	191 ± 8	0.52	0.405	0.743	0.158

^a^All *p* values obtained on univariate analysis. ^b^PDA, poorly differentiated areas. ^*∗*^Significant positive correlation of ER*α* expression and occurrence of PTCs in males. ^*∗∗*^Significant positive correlation of ER*α* expression and multicentric PDTC. ^*∗∗∗*^Significant positive correlation of ER*α* expression and incidence of distant metastases.

**Table 2:** Clinicopathologic correlation of ER*β*.

Sl no.	Attribute	ER*β* positive (*n* = 58)	ER*β* negative (*n* = 206)	*p* value^a^ overall	*p* value^a^ PTC	*p* value^a^ FTC	*p* value^a^ PDTC
1.	Age in years (mean ± SD)	45.9 ± 14.2	46.2 ± 15.9	0.865	0.371	0.707	0.086
2.	Gender (M : F)	19 : 39	71 : 135	0.876	0.305	0.507	0.014^*∗*^
3.	Presence of PDA^b^, *n* (%)	11 (24.4)	79 (43.2)	0.026^*∗∗*^	1.000	0.212	—
4.	Tumor size in cm (mean ± SD)	4.9 ± 3.1	4.6 ± 2.7	0.540	0.807	0.279	0.068
5.	Multicentricity, *n* (%)	15 (26.3)	55 (27.4)	1.000	0.679	0.668	0.389
6.	Extrathyroidal extension, *n* (%)	18 (31.0)	47 (23.3)	0.233	1.000	1.000	1.000
7.	Lymph node metastases, *n* (%)	31 (53.4)	71 (34.6)	0.014^*∗∗∗*^	0.836	0.117	0.310
8.	Metastases, *n* (%)	18 (31.0)	65 (31.9)	1.000	0.513	0.369	0.299
9.	Recurrence, *n* (%)	7 (12.0)	18 (8.7)	0.445	0.550	0.158	0.305
10.	Mean survival in months (*n* ± SE)	127 ± 8.5	197 ± 6.9	0.400	0.956	0.069	0.638

^a^All *p* values obtained on univariate analysis. ^b^ PDA, poorly differentiated areas. ^*∗*^Significant positive correlation of ER*β* expression and occurrence of PDTC in females. ^*∗∗*^Significant negative correlation of ER*β* expression and occurrence of PDA. ^*∗∗∗*^Significant positive correlation of ER*β* expression and incidence of lymph nodal metastases.

**Table 3:** Clinicopathologic correlation of HER-2.

Sl no.	Attribute	HER-2 positive (*n* = 19)	HER-2 negative (*n* = 245)	*p* value^a^ overall	*p* value^a^ PTC	*p* value^a^ FTC	*p* value^a^ PDTC
1.	Age in years (mean ± SD)	46.5 ± 15.9	46.3 ± 15.5	0.945	0.131	0.173	—
2.	Gender (M : F)	9 : 10	81 : 164	0.217	0.285	0.562	—
3.	Presence of PDA^b^, *n* (%)	3 (15.8)	87 (41.6)	0.762	0.514	0.261	—
4.	Tumor size in cm (mean ± SD)	4.9 ± 3.1	4.7 ± 2.8	0.027^*∗*^	1.000	0.014^*∗*^	—
5.	Multicentricity, *n* (%)	6 (31.6)	64 (26.8)	0.603	1.000	1.000	—
6.	Extrathyroidal extension, *n* (%)	7 (36.8)	58 (24.1)	0.269	0.358	0.266	—
7.	Lymph node metastases, *n* (%)	9 (47.4)	93 (38.1)	0.468	0.411	0.290	—
8.	Metastases, *n* (%)	3 (16.7)	80 (32.8)	0.195	0.186	0.219	—
9.	Recurrence, *n* (%)	2 (10.5)	23 (9.4)	1 : 000	1.000	1.000	—
10.	Mean survival in months (*n* ± SE)	151 ± 14.9	192 ± 7	0.792	0.090	0.391	—

^a^All *p* values obtained on univariate analysis. ^b^ PDA, poorly differentiated areas. ^*∗*^Significant positive correlation of HER-2 expression and overall tumor size; ^*∗∗*^FTC tumor size.

**Table 4:** Summary of the significance of ER*α*, ER*β,* and HER-2 expression.

Sl no.	Attribute	ER*α* expression	ER*β* expression	HER-2 expression
1.	Gender	PTC (male) 0.043^*∗*^	PDTC (female) 0.014^*∗*^	—
2.	Tumor size	—	—	FTC (large) 0.014^*∗*^
3.	Presence of PDA^a^	—	Whole cohort 0.026^*∗*^	—
4.	Multicentricity	PDTC (multicentric) 0.029^*∗*^ 0.037^*∗∗*^	—	—
5.	Lymph node metastases	—	Whole cohort 0.014^*∗*^ FTC (less) 0.023^*∗∗*^	—
6.	Distant metastases	Whole cohort (more) 0.04^*∗*^ 0.038^*∗∗*^	—	—

^a^PDA, poorly differentiated areas. ^*∗*^Significant *p* values on univariate analysis. ^*∗∗*^Significant *p* values on multivariate analysis. —, nonsignificant correlation. -Attribute within bracket- significantly more.

## Data Availability

The data are property of the institute and would be made available if specific request is made.
